# The feather’s multi-functional structure across nano to macro scales inspires hierarchical design

**DOI:** 10.1098/rsif.2024.0776

**Published:** 2025-04-23

**Authors:** Sebastian Hendrickx-Rodriguez, David Lentink

**Affiliations:** ^1^ Department of Mechanical Engineering, Stanford University, Stanford, CA, USA; ^2^ Faculty of Science and Engineering, University of Groningen, The Netherlands

**Keywords:** feathers, hierarchical materials, multifunctionality, bioinspired design

## Abstract

Bird feathers are finely tuned structures with key features at every length scale, from nanometre to metre, furnishing a unique multi-functional hierarchical design that can inspire material scientists, biologists and designers alike. Feathers are not only a crucial component in equipping birds with flight, but are also responsible for thermoregulation, coloration and crypsis, water repellency, silencing and sound production, sensing, directional fastening and even self-healing. Despite this broad multifunctionality, all feathers are formed from the same basic template using a universal building block: the feather keratin protein. Consequently, feather diversity across approximately 10 000 bird species arises from subtle differences in architecture rather than variations in chemical composition. To understand these underlying hierarchical mechanisms, we systematically review feather properties across all length scales, connecting development and morphogenesis to biomechanics and integrated structure–property–function relationships. This systematic distillation of the feather’s complex design into comprehensive principles will enkindle new biohybrid, biomimetic and bioinspired material solutions.

## 1. Introduction

After Robert Hooke examined feathers under the microscope in 1665, he delightfully reported a feather ‘contains neer a million of distinct parts, and every one of them […] adapted to a particular design’ [1]. Ever since, researchers have dissected the elaborate hierarchical architecture of feathers [2]. In particular how they grow and develop [3,4] to construct complicated architectures with enticing physical properties [5]. Feathers at their foundation are composed from limited but readily available chemical elements (e.g. C, N, O, H, Ca, P, S, Si) [6] that are arranged into complex hierarchical structures with multi-functional properties (at least 26 separate functions have evolved in modern birds) [7]. This holistic design approach, where the whole is greater than the sum of its parts, is a common motif for all natural materials [8]. Hence this review delineates our current understanding of the functional organization of the feather from the nanometre to the metre to emphasize hierarchical ramifications poised to inform future research and design across materials science, engineering and biology (for genetics and evo-devo also see [7,9]).

**Figure 1.**
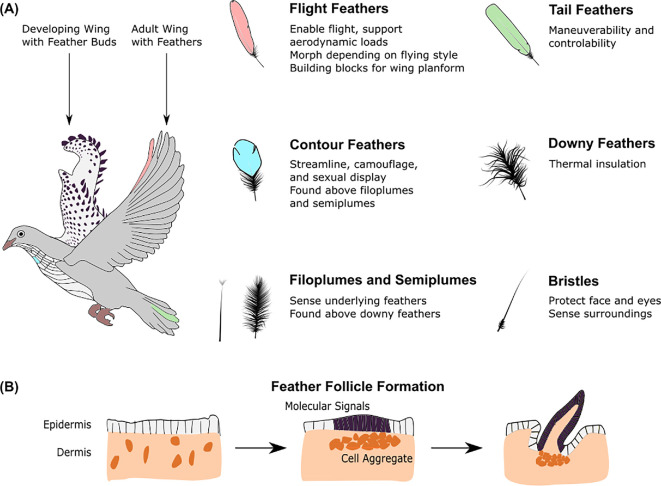
Bird feathers are designed for specific functions. (A) Six types of feathers cover the bird body at specific locations and perform unique functions. Wing flight feathers (shown in red) emerge from distinct feather buds (shown on the right wing) and withstand aerodynamic forces up to 10 g [10] without fluttering. Tail feathers (shown in green) form a precisely controlled tail that guides manoeuvres. Contour feathers (shown in blue) streamline the body and their colours serve camouflage, sexual display or thermoregulating functions. Downy (plumulaceous) feathers provide body insulation. Semiplumes and filoplumes are found beneath contour feathers, sensing the position of underlying downy feathers. Bristles (not shown on bird) protect the bird’s face and eyes [3]. (B) All feathers develop similarly. Sparse cells in the dermis contract to form aggregates that simultaneously direct cells in the epidermis to clump together (purple) [11]. Concurrently, molecular signals initiate differentiation of the cells in the follicle [12]. Cells proliferate at the base of the follicle, pushing old cells away to enable feather growth. Adapted from [11–13].

**Figure 2.**
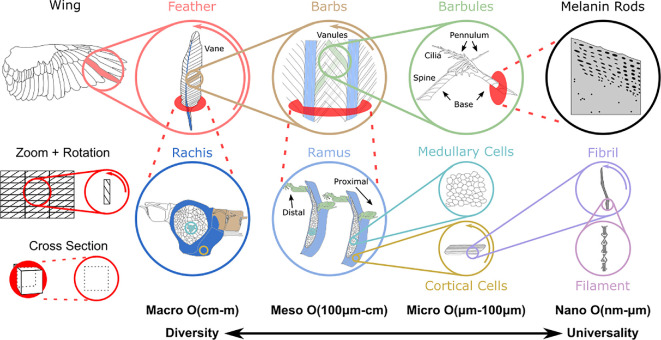
Feathers are hierarchically designed, multi-functional structural assemblies. Wings are a structural arrangement of flight feathers. At the macro scale, feathers consist of two aerodynamic vanes separated by a load bearing rachis. The feather branches on either side of the rachis into barbs that collectively form the two vanes of the feather. At the meso scale, barbs look like miniature feathers that consist of two vanules separated by a central shaft called the ramus. The barb branches on either side of the ramus into barbules that collectively form the two vanules of the barb. At the micro scale, barbules are column-like protrusions consisting of a base and pennulum with tiny outgrowths. Proximal barbules are characterized by a spine, while distal barbules have cilia hooklets. Furthermore, cross sections of the rachis and ramus reveal a pith-like core with medullary cells surrounded by cortical cells. Medullary cells have a vacuolated interior with circular shape. By contrast, cortical cells flatten out to form strand-like structures. At the nano scale, the inside of barbules is often filled with pigments such as melanin rods. By contrast, cortical cells and the periphery of medullary cells are formed by fibrils built up by a nano-scale filament of feather keratin protein. Adapted from [3,13,16,18,19].

A bird’s plumage consists of six basic feather types (figure 1A) with diverse features to control streamlining, insulation, communication, water repellency, crypsis, sound production and sensing [14]. Each type is found at a specific body location and fulfils a unique set of functions. However, feathers follow the universality-diversity paradigm [15] where this multifunctionality (diversity) is achieved by adapting the structural arrangement of a few (universal) constituents rather than inventing entirely new building blocks. For this reason, the growth of all feather types is the same with genetic expression at the molecular level and biomechanical stresses at the tissue level driving follicle development into hierarchical structures (figure 1B). Furthermore, the smallest building blocks of feathers, regardless of type or bird species, are keratin filaments at the nanometric protein scale [16,17]. Subtle differences in the arrangement of these universal units at larger scales, micrometre and up (figure 2), result in the multi-functional diversity of feathers. Each feather has unique properties and functions, but all follow a similar architectural template ranging from 10^7^ length scales for small birds such as hummingbirds and up to 10^9^ scales for large birds such as condors.

The multifunctionality of feathers is often a cumulative cascading effect from various length scales, therefore correlating particular structures to specific material properties is challenging. There does not exist a systematic framework for dealing with this structural complexity and the conventional tools used to measure mechanical properties of engineered materials are ill-suited for small, environmentally sensitive feather components. Therefore, many questions on the multi-functional nature of feather’s hierarchical organization remain.

This review provides a structural overview of the feather with an emphasis on how the structure naturally develops enticing properties by integrating length scales over nine orders of magnitude from nanometres to metres. We further discuss emerging functions from a biological, materials science, mechanical and biomimetic perspective—highlighting critical gaps in our understanding and emphasizing new opportunities for multi-disciplinary progress.

## 2. Keratin filament—nanometre scale

A keratin filament has a diameter of approximately 3 nm [17]. This molecular arrangement of atoms is the fundamental building block of feather structure and is found across all bird orders, providing a base level of strength, toughness, stiffness and durability. Investigating this protein is difficult due to its small size, but recent advancements provide insights into its formation, structure and properties.

### 2.1. Formation and structure

Keratin is a group of structural proteins found in the epithelial cells and integumentary system of many animals. Together with collagen, it is one of the important biopolymers in nature, crucial to the mechanical properties of epidermal appendages such as hair, wool, horns, scales and feathers [16]. There are two main types of keratin: α-keratins (intermediate filament or IF-keratins due to their 8−10 nm diameter) which form into an α-helix secondary structure, and β-keratins (also known as corneous β-proteins) which form into a β-sheet secondary structure. α-keratin is found in all vertebrates but β-keratin is exclusive to avian and reptilian tissues such as scales, claws, feathers and beaks [17]. Both α- and β-keratin are durable, tough and unreactive to the natural environment; they develop into a nanocomposite of crystalline filaments embedded in an amorphous matrix. Importantly, β-keratins form both the crystalline and amorphous halves of this composite without the need of other proteins—the same amino acid chain forms the filaments and the matrix [20]. This is a new paradigm when compared with engineered composites that require separate components for the matrix and filaments, often resulting in interfacial failure. This is also in sharp contrast with α-keratins, which only form intermediate filaments while the matrix is composed of keratin-associated proteins (KAPs) [20]. It is believed that β-keratins evolved in sauropsids as KAPs, but due to their unique biomechanical and morphological properties have taken over as the predominant protein [21].

Feathers are primarily composed of a β-keratin known as feather keratin. While there has been a recent push to rename this molecule as ‘feather corneous β-protein’ [21], in this review, ‘feather keratin’ is used for historical consistency. Feathers have traces of other β- and α-keratins (particularly in the calamus and rachis) [21,22], but most corneous material, especially in the barb and barbules, is feather keratin [21]. What is it in the chemical structure of feather keratin that makes it so unique?

**Figure 3.**
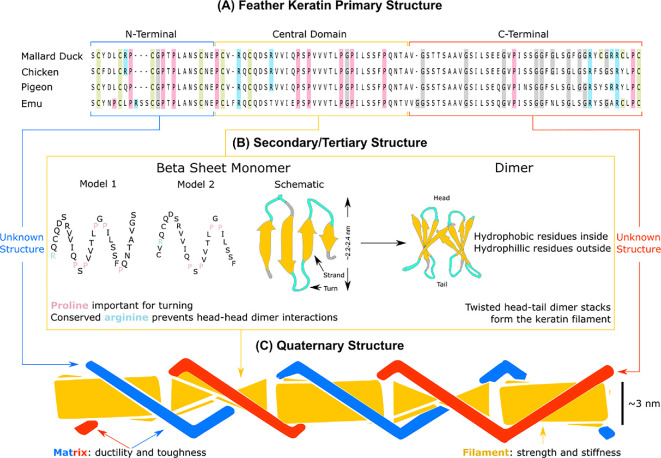
Feather keratin is the building block of feathers. (A) The amino acid chain of feather keratin is highly conserved across all bird species (exemplars shown) and has three regions: a nitrogen N-terminal, a central domain and a carbon C-terminal. Slight variations in the sequence are observed between feather types [22], though usually these mutations affect only a few amino acids in the C-terminal [23]. The terminal domains form an amorphous matrix responsible for the feather’s ductility and toughness, while the central domain forms crystalline fibres responsible for strength and stiffness. The conserved cysteine residues (light green) in the N-terminal form disulfide bridges that make the feather resistant to the outside environment. The conserved glycine residues (grey) in the C-terminal endow the feather with its elasticity. The central domain has two competing models [20,24] explaining how this region folds into fibres. (B) Both models predict a β-sheet monomer composed of successive antiparallel strands separated by hairpin turns. They both predict that proline residues (pink) govern hairpin turns, while a conserved arginine residue (blue) prevents head–head dimer interactions. A key difference is that Model 1 predicts five strands and four turns while Model 2 predicts four strands and three turns. The actual β-sheet monomers are amphiphilic with a hydrophobic and polar side. This enables two monomers to ‘sandwich’ together with their hydrophobic sides face-to-face to form a dimer. Twisted stacks of these dimers form the keratin filament. (C) This filament is represented as a stiff, yellow column stabilized by the matrix from the terminal domains shown as red and blue ropes. In the schematic, twisting angle is 90ᵒ which means four dimers stack before returning to the starting configuration. Different models predict different twisting angles. Adapted from [18,24].

The amino acid chain of β-keratins consists of three domains: the β-sheet forming central domain that folds into the filament framework, and the N-terminal and C-terminal domains that constitute the bulk of the matrix (figure 3A). A large part of the central domain is highly conserved, meaning nearly the same amino acid sequence (approx. 34 residues in length) appears in a wide variety of animal species and keratinous tissues [25]. Among avian feathers with known amino acid sequences, the emu feather shows the highest degree of variability in the sequence [26], perhaps because the emu is the only flightless bird examined so far, and the feathers of ground versus flying birds are under markedly different evolutionary pressures [27].

Feather keratin, unlike other β-keratins, also shows a high homology over its two terminal domains, further cementing the omnipresence of this unique amino acid sequence [26]. In the N-terminal domain, the high concentration of cysteine is thought to be involved in the intra- and inter-filament disulfide bonds that render feathers resistant to degradation. Furthermore, the N-terminal’s richness in charged residues such as cysteine and glycine is thought to increase the water content of the feather without compromising its insolubility, making the feather softer and more flexible. The C-terminal contains only one cysteine and thus has an insignificant role in the formation of the disulfide bonds [26]. However, the short length of this domain may help during the filament-forming process by enabling more direct interaction between monomers. In fact, proteins with longer C-terminals, such as the keratin found in claws and beaks, tend to form less organized bundles [21].

The central domain of feather keratin has been well studied and two models predict how it folds. Several authors inspected the central domain of β-keratins and concluded there are either five (Model 1) [20] or four (Model 2) [24] regions that have a well-defined tendency to fold into β-strands (figure 3B). Each strand is antiparallel to the one next to it and consecutive strands are separated by a hairpin turn. In both models, the hairpin turns are rich in proline, an amino acid that greatly affects protein shape [28]. Furthermore, the β-sheet monomer formed by the alternating antiparallel β-strands always has hydrophobic residues on one side, and polar groups on the other side [24]. The amphiphilic nature of this β-sheet secondary structure helps explain why β-keratins tend to self-assemble into organized hierarchical structures. Two completed β-sheet monomers distort and ‘sandwich’ together to form a dimer with hydrophobic surfaces face to face, while charged residues lie on the outer surface where they readily bind water. This dimer, formed out of a sandwiched pair of distorted β-sheets, interacts with other dimers to construct β-keratin filaments. The hydrophobic side-chains on the upper edge of one dimer, bind with the hydrophobic side-chains on another dimer’s lower edge. This ‘head–tail’ interaction is repeated several times to make the 3 nm diameter filaments characteristic of feathers (figure 3C). To avoid ‘head–head’ interactions detrimental to the filament-forming process, a conserved arginine is positioned externally to prevent the exposed edge of one dimer from inappropriately binding with the exposed edge of another dimer [20]. These keratin filaments, and their structure, govern the base mechanical properties of feathers.

### 2.2. Mechanical properties

Due to the omnipresence of the feather keratin amino acid sequence across bird orders, all species are equipped with a base level of mechanical properties. It is clear that the molecular structure of feather keratin makes it resilient to all but the most violent proteolytic enzymes, chemicals and degradation conditions, resulting in 65 million tons of annual feather waste from the poultry industry [29]. This same resilience makes feather keratin a prime target molecule for palaeontological studies of fossils [30]. The strength of this molecule stems from the disulfide bonds formed by cysteine in the N-terminal domain. The energy needed to break one of the bonds that holds feather keratin together—also known as the activation enthalpy and measured for peacock feathers—is 172 ± 86 kJ mol^−1^ [31,32]. The bond strength of a covalent disulfide bridge S–S (approx. 215 kJ mol^−1^) fits well in this range. Furthermore, the molecule remains robust throughout the entire feather length, demonstrating incredible consistency in the manufacturing process despite occurring inside a chaotic cell environment over several days to months [18]. This folding process is not trivial as researchers have tried disentangling feather keratin to spin new fibres, yet the regenerated structures have markedly lower tensile strength than that of raw feathers [33].

This shows that innovation is still needed to develop equipment and theories that characterize the smallest scale of feather organization. Till now, most keratin research has been dedicated to α-keratin found in mammal tissue [17], leaving questions about the mechanical nature of β-keratin found in reptile and avian tissues. The two terminal ends of the feather keratin protein have been particularly understudied; their folding pattern, function and importance remain unknown. However, current knowledge indicates that the mechanical properties and structural organization of feather keratin remain largely unchanged throughout the feather. Therefore, multi-functional diversity among feathers cannot solely come from this protein at the molecular level. Instead, diversity is built in at larger length scales.

## 3. Cortical and medullary cells—micrometre scale

All feathers can be described as the methodical arrangement of cells, where feather morphologies (figure 1) stem from organizational differences during development. Two types of cells form feathers: (i) long, fibre-like cortical and barbules cells forming the hard cortex exterior, and (ii) circular, vacuolated medullary cells shaping the pith-like core interior of the rachis and ramus. The combination of both cells integrated as a sandwich-structured composite explains why feathers are both strong and light. At this level of functional organization, we examine developmental biology to connect molecular activity and cellular behaviour to morphogenesis of the feather at the organismal level.

### 3.1. Development

**Figure 4.**
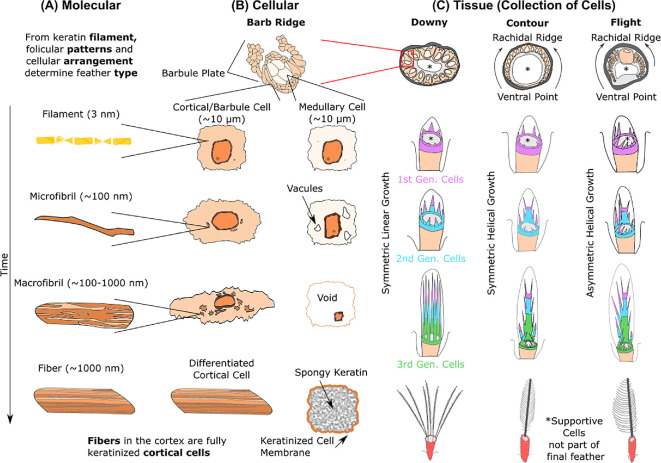
Feather maturation across subcellular/molecular, cellular and tissue/organismal levels. This figure shows development at each level with early stages at the top and finalized stages at the bottom. (A) The continuous production of 3 nm feather keratin filaments results in an aggregation of these components into larger and larger fibrillar structures from microfibrils, to macrofibrils, to fibres. Approximate diameter of these fibrillar structures are shown. (B) A barb ridge consists of medullary cells surrounded by cortical cells with two arms or plates of barbule cells branching out. All cells are of the order of tens of micrometres in size, though the size of the barb ridge varies with bird species. Birds modulate the barb ridge size by the number of cortical, barbule and medullary cells included in the arrangement. Cortical and barbules cells synthesize keratin filaments that initially accumulate along the periphery, but soon expand into the entire cytoplasm as the cell elongates and becomes fully keratinized. Medullary cells only aggregate filaments along their periphery while the cytoplasm develops large vacuoles, empty of subcellular organelles, that merge into a singular void which rapidly phase separates into a substance known as spongy keratin. (C) Differing sizes, arrangements (symmetric versus asymmetric) and growths (linear versus helical) of barb ridges result in different bird feathers. For downy feathers, barb ridges are circularly placed around the follicle and grow straight up resulting in unconjoined barbs. For symmetric contour feathers, barb ridges grow helically away from a ventral point before merging at the dorsal point to form the rachidal ridge. The ventral and dorsal point are polar opposites for contour feathers—changing this orientation gives rise to asymmetric flight feathers. Purple represents the first ‘generation’ of cells placed during growth, blue the second, and green the third. The base of the feather follicle is shown in beige. Adapted from [21,35,36].

Feather development begins at the feather follicle where the epidermis folds inward around the dermal papilla, an extrusion from the dermis [34] (figure 1B). The epidermis surrounding the dermal papilla is known as the germinal collar and is constricted at the bottom. Stem cells proliferate at the base of the collar, pushing older cells distally as newer cells take their place. As cells migrate, they differentiate into either cortical/barbule or medullary cells. Cortical/barbule cells differentiate in a process known as keratinization, while medullary cells undergo vacuolization. Keratinization involves the aggregation of feather keratin filaments to form larger and larger bundles [21,34] (figure 4A). This review uses the historical name ‘keratinization’, though the process is sometimes referred to as ‘cornification’.

Both cortical/barbule and medullary cells begin as undifferentiated stem cells, of a few to tens of micrometres in diameter (figure 4B). Inside cortical/barbule cells, keratin filaments begin aggregating as the cell elongates to form a narrow, tube-like shape. Since keratin filaments are surrounded by a glue-like matrix that completely enshrouds them (figure 3C), this aggregation process could only be studied by X-ray diffraction [37–41] until staining and electron micrography provided visualization of the keratin filaments [42,43]. Larger fibrillar structures could only recently be observed by using microbes to dissolve the matrix [44,45].

We propose a nomenclature, based on the fibrillar structure of collagen and α-keratin, to standardize the different hierarchical levels (figure 4A) starting with the 3 nm *filaments*. Clusters of these feather keratin filaments spanning to around 100 nm are named *microfibrils*. During keratinization, the cortical cell begins accumulating these microfibrils along the periphery of the cell to form *macrofibrils* of a few hundreds of nanometres (100−1000 nm). At the same time, all other organelles degenerate and disappear, including the nucleus. Eventually these macrofibrils fill up the entire cell, measuring a few to tens of micrometres. We distinguish this differentiated cell by calling it a *fibre*.

Instead of aggregating bundles of keratin, medullary cells develop large vacules in their cytoplasm, restricting keratin to the periphery of the cell [46]. Small vacules merge to form a single large void that occupies most of the cell, pushing the nuclei and other organelles to the cell membrane, which at this point starts to keratinize. Once the cell membrane forms a solid layer of keratin, approximately 750 nm thick, a spongy lipid substance appears inside the void [46]. The spongy keratin phase separates from the cytoplasm in one of two ways: spinodal decomposition or nucleation-and-growth. In the former, it separates from the cell cytoplasm spontaneously to grow and form tortuous channels. In the latter, separation starts at random nuclei points throughout the volume to form isolated spherical cavities (electronic supplementary material, §2, figure S2C). The precise mechanisms through which these nanostructures self-assemble is still unknown, but depend on the interplay between entropy and molecular interactions. In spinodal decomposition, there is no activation barrier to separation and the solution demixes smoothly throughout. In nucleation and growth, an activation energy barrier stabilizes the solution until the minority phase aggregates into a particle of some critical size. The rate of spongy keratin expression, spongy keratin polymerization and filament cross-linking are all thought to play a key role in determining which of the two processes occur [47,48].

Some cells support the feather structure during growth but degenerate once development is complete. These supportive cells passively carve out the branching of barbs [49] and help mould the hooks, cilias and spines that characterize barbules. Also important is the presence of α-keratin in the feather follicle before feather keratin appears [35,50], because absence of this molecule produces feathers with a twisted rachis resulting in frizzled plumage [51].

With the basic cell types and their growth timelines identified, we discuss how changing the arrangements of these basic components produce different feather types (figure 4C). For example, the coat of precocial chicks consists of downy feathers that have unconjoined barbs forming a loose, fluffy layer. These individual barbs start as barb ridges (figure 4B) that consist of medullary cells surrounded by cortical cells, with two extending ‘arms’ known as barbule plates (made of barbule cells). Multiple barb ridges arranged in a circle, with supportive cells filling the remaining space, result in unconjoined barbs. Specifically, new cells proliferate directly beneath older cells that differentiate as they are pushed distally, resulting in a linear growth path to build long unconjoined barbs (figure 4C—Downy). Once development is complete, the unconjoined barbs consist of a medulla surrounded by a cortex, with barbules branching out periodically.

During moulting, reactivation of stem cells at the base of the feather follicle results in the formation of more complex pennaceous feathers such as contour, flight and tail feathers. In the case of the symmetric contour feather, initial arrangement of cells is like the downy feather with barb ridges forming a large circle. In contrast to downy feathers, the proliferating cells of the barb ridges do not divide directly beneath older cells. Rather, they gradually shift away from a ventral point on the circle converging at a dorsal point 180° away. This results in a helical growth path with formation of a rachidal ridge at the dorsal point due to progressive fusion of barb ridges from the left and right [52] (figure 4C—Contour). Asymmetric flight feathers form by changing the relative positions of the ventral and dorsal points; barb ridges move different distances on either side of the ventral point before fusing into longer or shorter barbs [21,53] (figure 4C—Flight). The exact way how barb ridges fuse to form the rachidal ridge remains unclear; however, the rachidal ridge clearly shows a large grouping of medullary cells surrounded by cortical cells destined to become the rachis medulla and cortex, respectively. Other feather types such as bristles, filoplumes and semiplumes can be formed by activating/deactivating the emergence of barb ridges and controlling whether they grow linearly or helically [54], though the exact mechanism remains unknown. With this conceptual framework of the biological development of cortical and medullary cells, we can assess how their assembly yields exceptional mechanical properties.

### 3.2. Structure and properties

**Figure 5.**
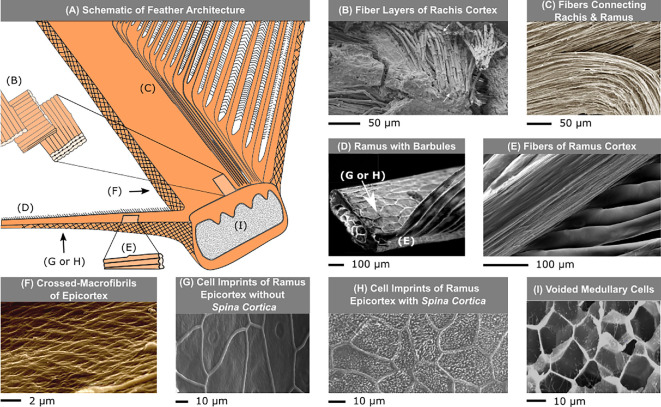
The cortex and medulla are two fundamental, multi-functional, complementary components that make up the sandwich structure of a feather. (A) A schematic feather is shown emphasizing the various structures of the cortex and medulla. The dorsal (topside) and ventral (underside) rachis cortex is made of (B) three fibre layers, of varying thickness and fibre orientation [55]. (C) Only the longitudinally oriented fibres diverge from the rachis and follow the (D) ramus away from the centre of the feather [56]. (E) Longitudinally oriented fibres are seen on the top surface of the ramus cortex [57]. The lateral walls of the cortex, known as the epicortex, are made of (F) layers of diagonal crossed-macrofibrils that connect the barbs to the rachis [58]. The epicortex flows naturally from rachis to ramus. On the ramus epicortex, cell boundaries brand the surface with several ridges. (G) For some birds, the cell is bare except for small craters where cell nuclei used to be. (H) For other birds, the surface is covered with tiny thorn-like protrusions referred to as *Spina cortica* [59]. (I) Electron micrograph of spongy cells that make up the medulla. A few, spider-like microfibril threads sprawling across the cell interior are nearly indiscernible [57]. Adapted from [58]. Reprinted from [44,56–58] with permission. More electron micrographs of different species available from [60,61].

The cortex and medulla (figure 5A) have evolved to be multi-functional and play key roles in processes from toughening the feather for flight to creating brilliant, iridescent colours for communication. The cortex is a fibre-reinforced, laminate composite material that encircles the medulla of both the rachis and ramus [62]. The dorsal (topside) and ventral (underside) rachis cortex (figure 5B) is made of three layers of long (tens of micrometres) fibres deemed syncytial barbules, covered in a matrix glue [44]. This is different than the filament-matrix nanocomposite of feather keratin protein. The lateral walls of the cortex are covered by a more ductile layer, known as the epicortex, made of layers of diagonally crossed macrofibrils [58]. Fibre connections are mediated by thickened nodes, remnants of barbule cell junctions. These near-periodic syncytial nodes are staggered in three dimensions in the cortex resulting in a ‘brick-bridge-mortar’ structure that slows crack propagation (electronic supplementary material, figure S2A,B). These nodes have hooked ends to latch on to other fibres, preventing ‘pull-out’ of fibres from the surrounding matrix [44].

Observed in several bird species, the innermost layer of the rachis cortex closest to the medulla, comprises fibres longitudinally oriented with the rachis (approx. 0–10° to the rachidal long axis), making up 80–85% of the thickness of the cortex. A zone of circumferentially oriented fibres (approx. 60–70° to the rachidal long axis) lies directly above and makes up approximately 15% of the thickness [44,58]. A top layer just a few cells deep consists of longitudinally oriented fibres, perhaps to streamline the rachis surface [55]. Longitudinal fibres in the rachis cortex diverge uninterrupted into the ramus (figure 5C), providing a continuous attachment and distributing load from slender barbs to the rachis, while gradually reducing rachis volume in the proximo-distal direction. This avoids abrupt fibre terminations that would concentrate stress at discontinuities, a potentially catastrophic crack-defect for feather integrity. The predominantly axial orientation of fibres in the rachis and ramus (figure 5D,E) helps maximize flexural rigidity (resist bending) [58]. However, this leads to toughness anisotropy (measured in ostrich feathers) with the longitudinal cutting energy (approx. 5 ± 1 kJ m^−2^) one third the transverse energy (approx. 15 ± 2 kJ m^−2^), because in the former the cut is parallel to the cortical fibres while in the latter the cut is across fibres [63]. Composites with only unidirectional fibres have low torsional stiffness, which could be detrimental to flight efficiency if the rachis or ramus twist too much [55]. Therefore, the rachis and ramus also have an epicortex to increase their torsional stiffness and strength.

The epicortex does not contain large fibres but consists of alternate layers of oppositely oriented macrofibrils (100−1000 nm in diameter) approximately 45° to the rachidal or ramus long axis (figure 5F), making it less brittle than the cortex. Barbs and barbules attach to this more ductile surface [45,58]. The orientation of these macrofibrils helps resist shear stresses which are greatest at ±45° to the long axis if the feather cortex is considered as a thin-walled cylinder system [64]. The side surface of the ramus cortex tends to be rough due to the impressions of the cell nuclei, cell boundaries and coarse fibrillary striations (figure 5G,H). In a subset of birds, small thorn-like microstructures grow perpendicular to the surface of the ramus cortex, reminiscent of microvilli but fully keratinized. Sometimes these grass-like structures, deemed *Spina cortica*, were so abundant that they formed a cuticle lawn named *Tapetum spinosus* similar to a brush border. These surface structures may function as dampers for unsteady loading on the ramus cortex [59]. However, it remains unclear why variations in surface roughness occur across orders, what their purpose is, or which birds possess these structures [56,57,60,65–68]. Micro-texture could dictate the reflective properties of the surface, while nano-texture could play a role in structural colour [69].

The medulla is composed of vacuolated cells with keratinized boundaries. Electron micrographs show the effervescent nature of the medulla, with crystalline macrofibrils cell membranes interweaving around spongy feather keratin (figure 5I). Sometimes keratin microfibrils interconnect opposing boundaries like a cobweb covering, acting as colour-producing structures for some birds [46]. Structural and mechanical properties of medullary cells both in the rachis and ramus remain understudied. However, it has been shown that cell size, elongation and orientation are carefully controlled in the medulla to support different modes of flight in various bird species [70].

Even though the mechanical response of all feathers is dictated by the material properties and structural combination of the cortex and medulla, only a few measurements on a limited number of species have been made on their mechanical properties. These measurements have primarily been of simple parameters such as Young’s modulus (stiffness or a quantification of how much a material resists deformation in response to an applied force) and ultimate tensile strength (a measure of how much stress a material can withstand before permanently deforming). So far, measurements reveal broad consistency in cortex keratin’s elastic properties across bird orders (including ratite and carinate) and feather type (tail, contour and flight). The tensile strength and stiffness of the cortex is comparable to engineering polymers such as polyesters and epoxies (table 1). By contrast, the compressive strength of the medulla—measured for peacock feathers—is approximately 0.5 MPa when dry and approximately 0.1 MPa when wet (tests ran at a strain rate of 1 × 10^–3^ s^−1^ or approximately 0.3 mm min^−1^ for the size of the samples used) [75]. Young’s modulus of the medulla—measured in compression for swan feathers—ranges between 6.7 and 35.3 MPa (at a test speed of approx. 7 × 10^−3^ s^−1^ or 1 mm min^−1^) [76] and scales roughly quadratically with medulla density, $\rho_{medulla}$, which ranges from 0.046 to 0.086 g cm^−3^. The theory of cellular solids predicts that a foam’s modulus should scale with medulla density as $E_{medulla}\propto E_{keratin}{\left(\rho_{medulla}/\rho_{keratin}\right)}^{n}$ where *n* is between 2 and 3 [77]. The density of compact keratin found in the cortex and medullary cell boundaries is $\rho_{keratin}$ = ~1.15 g cm^−3^. Because of its low modulus, the medulla plays an insignificant role in determining the flexural stiffness of the feather rachis and barbs. However, removing this low-density foam interior and leaving only the hollow thin-walled cortex, would greatly expedite the onset of local buckling. In fact, the medulla acts as an energy absorber in transverse compression of the composite rachis as 96% of the stored elastic energy is found in this foam structure [78].

**Table 1.** Tensile tests have been performed on cortex pieces of several species from different feathers and at varying environmental conditions. The table shows a list of these tests from least stiff to most stiff. Observations: (i) relative humidity has a large effect on strength and stiffness, with ostrich contour feather values more than doubling from 100% to 0% RH, and (ii) wing feathers may have a slightly lower stiffness in order to reduce stresses during flight. Reported values are complemented by the standard error of the mean (s.e.m.) across samples tested.

modulus (GPa)	strength (MPa)	species/material	feather type	RH (%)	temp. (°C)	strain rate (mm min^−1^)	reference
~0.01–0.1	~0.1−10	polymeric foams	—	—	—	—	[8]
~0.1−10	~10–100	engineering polymers	—	—	—	—	[8]
1.5 ± 0.1	106 ± 11	ostrich	contour	100	room	1	[71]
1.7 ± 0.4	—	ostrich	wing	75	23	1	[72]
1.8−2.8	—	various	primary	room	room	1	[73]
2.4 ± 0.5	—	ostrich	contour	75	23	1	[72]
2.6 ± 0.2	130 ± 14	ostrich	contour	50	room	1	[71]
2.6 ± 0.1	78 ± 6	toucan	tail	—	—	1.14	[74]
3.7 ± 0.3	221 ± 18	ostrich	contour	0	room	1	[71]
~10–500	~10–2000	engineering alloys	—	—	—	—	[8]

To fully comprehend the intricate composite microarchitecture, the mechanical properties of both the cortex and medulla need to be fully characterized and compared phylogenetically across species to identify functional adaptations. This is challenging due to mechanical time- and temperature-dependencies, and because the dimensions of the test sample need to be sufficiently larger than the (minimal) critical size at which meso-structural features affect mechanical behaviour [73,74]. This means that the experimental measurements that are particularly well suited for classic, homogeneous materials such as metals, only serve as a coarse step in characterizing hierarchical biological materials such as feathers. Furthermore, these mechanical measurements can only be placed in a biologically meaningful context if the bird’s natural habitat is carefully considered when setting laboratory experimental or computer simulation conditions (humidity, temperature, strain rate, etc.). This is essential for phylogenetic comparisons across bird orders. To place measurements of these individual features in the context of the entire feather structure, we next examine another micro-scaled component: the feather barbule.

## 4. Barbule—micrometre scale

The cells in the barb ridge that form barbule plates (figure 4B) transform into barbules: long column-like protrusions with micrometre diameter composed of a base and pennulum that extend from the ramus to form vanules. Proximal barbules are characterized by a long spine at their base, and distal barbules by hooked cilia on their pennulum. Flight feathers acquire their smoothness when hooked cilia latch on to the spine of a proximal barbule, helping hold the feather together. How are barbule structure–property–function relations developed and how are barbules distributed in a feather?

### 4.1. Development and structure

Barbules are the final differentiation state of barbule cells and develop in a similar way to cortical cells. Unlike cortical cells that remain inside the cortex as fibres, barbule cells are surrounded by supportive cells that fully degrade and vanish as the feather develops. Though supportive cells are critical for shaping the barbule, they play no role in final feather function. Because of the disappearance of supportive cells, barbules are free to interact with the outside environment and due to this accessibility, have been studied extensively.

**Figure 6.**
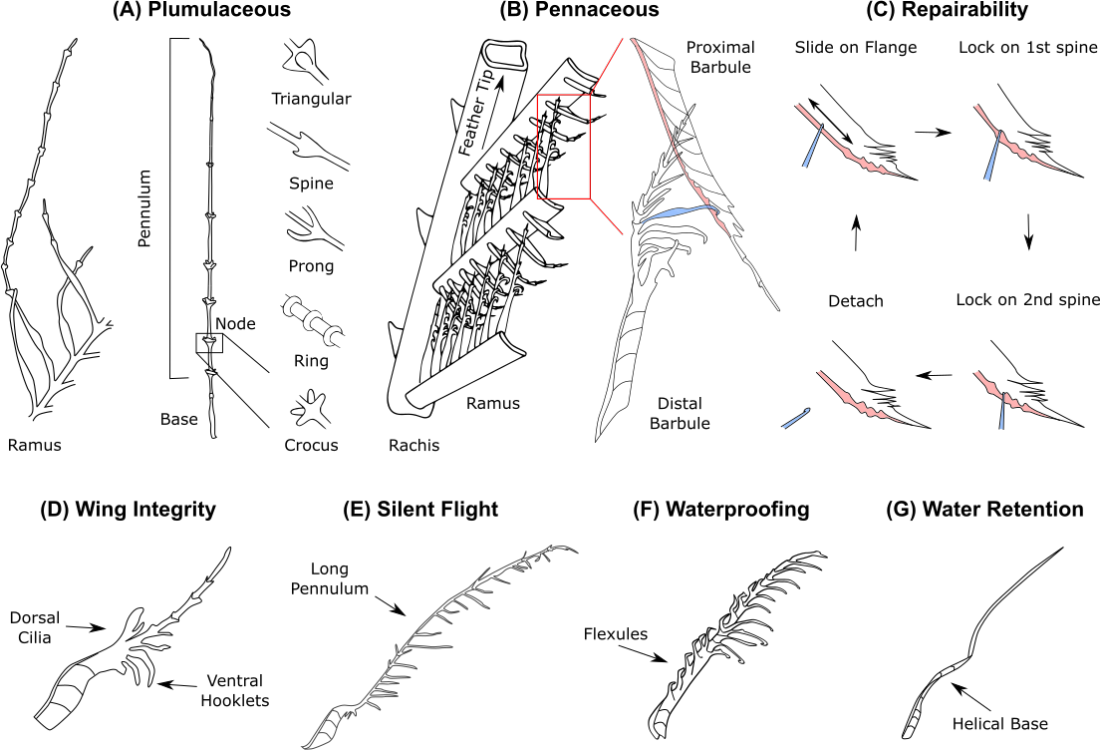
Barbule shape is generic but the functional morphology varies with species, feather type and feather vane location. Barbule structural diversity enables specialized functions. (A) For plumulaceous feathers without central rachis, barbule bases extend from the ramus and continue into one long pennulum. These telescoping pennula have different types of nodes depending on bird species and are forensically useful [79]. (B) For pennaceous feathers where rami extend from a central rachis, there are two different types of barbules depending on what side of the ramus they extend from. Distal barbules point from feather base to feather tip and are characterized by ventral hooklets (coloured in blue). Proximal barbules point from feather tip to base and are characterized by dorsal flanges with small bumps (coloured in red). (C) Hooklets from distal barbules slide along this proximal barbule flange during feather unzipping, before being locked into place by one of the many dorsal spines. If this barbule pair detaches, birds can easily repair connectivity, e.g. by running a beak through their feathers. (D) Dorsal lobate cilia help maintain wing planforms by interacting with rami of other feathers. (E) Long pennula on owl feathers help silence aerodynamic and feather sliding noise during flight. (F) Flexules found on barbule bases of aquatic birds such as grebes and cranes create a porous surface through which water cannot enter due to surface tension. (G) By contrast, desert sandgrouse belly feathers possess barbules with helical bases. When wetted, these bases uncoil (possibly due to variations in keratin crystallinity) and the long, straight pennula rotate up out of the initial plane of the vane. These pennula bend into tufts that retain water as the bird flies towards its nest, away from watering holes [80]. Adapted from [3,79–83].

Barbules can roughly be described as columns of cells, with connections from one cell to another known as nodes (figure 6A). All barbules are composed of a base of fused cells that connects it to the ramus, and a telescoping pennulum where nodes are easily observed. Unlike cortical fibres where node shape was generally homogeneous, barbule nodes can take on a variety of shapes depending on species, age, feather type and location on the feather [79].

Proximal and distal barbules are responsible for keeping flight feathers smooth and intact (figure 6B) [81]. Distal barbules are characterized by many hooklets protruding from the pennulum on the ventral side (underside) of a feather. They are located on the side of the ramus facing the feather tip and point distally away from the feather base. Proximal barbules look like curved sheets with dorsal (topside) flanges that have sawtooth bumps near the barbule tip. They are located on the side of the ramus facing the feather base and point proximally towards the feather base. This pair of barbules act as a repairable cascaded slide-lock system composed of hooklets and a slide rail or flange [81]. Hooklets freely and reversibly slide along this flange until reaching sawtooth bump terminating structures, known as dorsal spines, which lock hooklets in place and keep the feather vane interconnected (figure 6C). If enough force is applied to detach a hooklet from the first dorsal spine, there are more spines further along the flange to lock it again. The separation force required to fully displace a distal barbule from its place next to a proximal barbule, known as unzipping, remained nearly unchanged after 1000 cycles of separation and repair (approx. 44 ± 9 mN) [81]. The shapes of distal barbule hooklets have evolved to strike an optimal balance between strength of hooking and ability to re-hook [84]. Plumulaceous barbules of downy feathers do not have this detailed structure and therefore do not lock together.

Aerodynamic constraints drive microscopic functional morphology: variation in barbule shape can be examined between species, across a wing and even over a single feather. For example, some distal barbules bear lobes on their dorsal side (figure 6D) that interact with the ramus of other feathers, creating mechanical fastening zones between two adjacent overlapping feathers to form a continuous wing planform [3,85,86]. Termed ‘directional Velcro’, these distinctive underlapping three-dimensional hooking microstructures probabilistically fasten on to overlapping two-dimensionally hooked rami during wing extension (when the wing is spread out), and automatically unlock during wing flexion (when the wing is tucked in) [87]. This unique probabilistic mechanical fastener is secure, reversible and repeatable, but also makes a Velcro-like noise [88]. Consequently, silent fliers lack them. Instead, elongated pennula create a velvet-like feather surface that makes owl flight extremely quiet (figure 6E). These pennula reach their full length only in wing regions where flow separation occurs [89]. In aquatic birds, curved dorsal fibres known as flexules are found on the dorsal edge of proximal and distal barbules (figure 6F), creating a porous surface through which water cannot enter due to surface tension [83]. In desert sandgrouse, helical barbules (figure 6G) uncoil upon wetting, resulting in pennula that point perpendicular to the feather plane. These pennula clump together to form teardrop-shaped tufts that can retain water [82]. As water has been shown to self-heal feathers (electronic supplementary material, §3), it is interesting to consider how birds living in wetter or dryer habitats have evolved. While some sea birds are frequently exposed to water, others only encounter moisture when it rains.

To fully understand the functional morphology of barbules, we must consider how they function together in a vanule. A single ramus may have hundreds of barbules protruding from both sides, shaping the vanule by altering barbule length, barbule-barb angle and protrusion location. How do these morphological features differ between bird families and ecological and flight specializations, and perhaps more importantly, what stays the same?

### 4.2. Distribution and function

**Figure 7.**
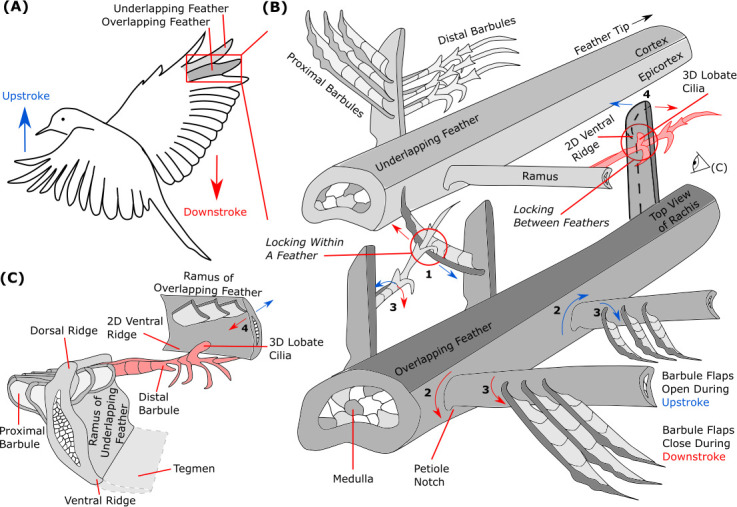
Hierarchical interactions within the feather vane and between adjacent overlapping feathers furnish a continuous surface that can open and close. (A) Structural features at different length scales can modify the aerodynamic surface formed during every wing upstroke (blue) versus downstroke (red). (B) Barbs within a feather vane strongly lock together in a Velcro-like fashion when the hooks of distal barbules slide outward on the spines of proximal barbules during the downstroke; the hooks slide inwards during the upstroke (shown next to **1**). Furthermore, barbs are obliquely attached to the rachis at the petiole notch; the dorsal ridge of a barb is closer to the feather tip than the ventral ridge of the same barb. This is believed to cause proximal rotation during the downstroke to lock hooks on to spines, and distal rotation during the upstroke to release hooks (shown next to **2**) [83]. Adding to this asymmetric response, putative barbule flap rotation forms a unidirectional valve that opens during upstroke and closes during downstroke (shown next to **3**). Between feathers, one-way locking ‘directional Velcro’ activates during the downstroke, but unlocks during the upstroke. Locking occurs when one of the tens to hundreds of two-dimensional (2D) hook-shaped ventral ridges of the overlapping feather’s rami latch on to one of the thousands of three-dimensional (3D) hook-shaped dorsal lobate cilia of the underlapping feather’s distal barbules (shown next to **4**). (C) Cross section of two feathers shows the directional probabilistic locking mechanism between two-dimensional hooked rami (overlapping feather) and three-dimensional hooked lobate cilia (underlapping feather). The barb cross section also illustrates how the extended ventral ridge forms a long tegmen in waterfowl, which is thought to make the wing airtight under elevated loading [5]. Adapted from [3,80].

The structure of barbules varies pronouncedly between different bird species to furnish a wide range of functions, each suited to a particular ecotype. However, the distance between adjacent barbules protruding from the ramus of a flight feather ranges only from 8 to 16 µm for a diverse set of birds for which this has been measured—from the Anna’s hummingbird (*Calypte anna*) to the Andean condor (*Vultu gryphus*) [84]. This distance might be slightly larger for proximal barbules than distal barbules [3] as well as for non-flight feathers [90]. While more research is needed to fully understand this scale invariance, the most parsimonious explanation is that barbule spacing must be in this narrow range to limit permeability of air through the feather vane [84]. Since lift scales with non-porous feather area, if this spacing were to increase proportionally to bird size, larger birds would be unable to generate the lift needed for flight due to leaky surfaces. On the other hand, reducing this spacing might limit the torsional flexibility of barbules and increase the amount of keratin material that needs to be developed (figure 7).

The way barbules project from the ramus to form the vanule dictates how proximal and distal barbules interact during flight and maintain the structural integrity of the feather. For example, proximal barbules curve near their tip, ensuring feather cohesion throughout flight [80]. To further safeguard against unzipping, barbules are sufficiently compliant to bend slightly once proximal hooklets have been locked on to a dorsal spine. This assures barbules stay attached rather than fail [84].

Arguably the most important aspect of barbules is how they interact with fluids such as air and water during flight. The drag force of a feather decreases as its barbules become more disengaged (less connections between proximal and distal barbules). According to experiments with 3D printed model feather vanes, overlapping membranous side flaps located on barbules may also act as unidirectional valves (figure 7), allowing airflow in the dorsal direction (during the upstroke) but not in the ventral direction (during the downstroke) [84,91]. For aquatic birds, barbules trap air between them (like flexules in figure 6F) creating a thin layer known as a plastron that inhibits wetting. This layer also enhances insulation and provides additional buoyancy to birds [92].

Knowledge gaps related to barbule form and function fall into two categories: barbule formation during development and taxonomic functional design differences. How is the broad range of barbule morphologies achieved, both their external shape and internal structure? How are these different morphologies mechanistically advantageous to the birds that possess them? Did environmental and physical constraints drive the evolution of these diverse adaptations? If so, we expect to see similar structures in birds living in similar ecologies, even if they come from different orders. Answering these questions methodologically, by harnessing the avian phylogenetic tree, can help us understand how barbules ended up integrated with other microscale features to form even larger, hierarchical structures across evolutionary timescales [7].

## 5. Barb—millimetre scale

Collectively, barbs form the largest part of a pennaceous feather by area: the feather vane. Given the structural prominence of the barb, it is important to determine its precise shape and how this structure imparts mechanical properties. How have these physical traits been fine-tuned to best adapt to a bird’s ecosystem and aeromechanically support flight?

### 5.1. Shape and structure

Barbs are the smallest example of a holistic structure that combines cortical, medullary and barbule cells. At first glance, barbs appear like miniature feathers complete with a hard cortex and vacuolated medulla (forming the ramus), and diverging branches (i.e. the barbules). However, this view misses the complexities that enable hundreds of barbs to come together and form the feather vane, which provides the bird with a strong aerofoil for flying [3]. To comprehend this, barb shape and structure need to be analysed holistically, including the way they extrude from the rachis, and how this morphology affects the barb’s mechanical properties.

The ramus of a barb is asymmetrical with a convex proximal side and concave distal side (figure 7C), though it gradually tapers off and becomes more symmetrical towards its tip. The topside and underside of the ramus are known as the dorsal and ventral ridges, respectively. The cortex walls are thicker than corresponding lateral sides because of higher loading in the dorsal–ventral direction during flight, and because the ramus is able to twist along its lateral sides when critical loads are applied [93]. Since the critical buckling stress scales with the square of beam’s slenderness ratio $\left(\sigma_{crit}\propto (L/r{)}^{2}\right)$, these shorter lateral sides are much more likely to fail by buckling during bending. However, it has been suggested that this may be advantageous, as failure by buckling is less catastrophic than failure by rupturing, which would occur if the critical buckling stress was larger than the yield stress of keratin [94]. An extended ventral ridge, known as a tegmen (figure 7C), is observed in several species of waterfowl (ducks, geese and swans), and appears macroscopically like a glazed, shiny surface extending near the rachis [3]. The tegmen is believed to act as a flap valve, preventing air from passing upwards between barbs [5]. Small notches can sometimes be observed in the ventral border of a ramus just before it merges with the rachis; these are known as petioles. These petioles might enable ramus twisting during the upstroke and downstroke (figure 7B), acting similarly to plant petioles which facilitate reorientation of leaves towards the downwind side [95]. Notably, the medulla of the ramus starts beyond the petiole and is not continuous with the medulla of the rachis [3]. The ratio of cortex to medulla area changes along the ramus in a manner that depends on species. Generally, the ratio decreases slightly after the ramus base, but increases steadily after reaching the midpoint of the ramus [93]. Since flexural rigidity of a beam (D=EI) depends on the Young’s modulus (E) and second moment of area (I), increasing the ratio of stiff cortex to compliant medulla could help counteract tapering of the barb. Thereby, maintaining flexural rigidity throughout the barb length.

Barb angle and length determine vane shape which affects the final aerodynamic properties of a feather [96]. Barb length is controlled by the relative position between the rachidal ridge and ventral point during development. Barb length increases at the base of the feather, then remains constant before decreasing at the feather tip. Due to barb length asymmetry between the two vanes in a flight feather, the rachis is not positioned in the centre of the feather. Instead, it is closer to the feather side that first contacts air, known as the leading edge. This asymmetry stabilizes feather pitch during flight, preventing flutter. Furthermore, feather vane emarginations at the wing tips, resulting from rapid changes in barb length, reduce the induced drag on the wing. This ‘tip sail’ function is analogous to the single winglets on modern passenger aircraft [97]. Barb angle is controlled by differential expansion as the feather unfurls from the sheath after development, with smaller angles resulting in a stiffer vane [96]. Barb angle steadily diminishes from base to tip for primary flight feathers and outer tail feathers, but remains unchanged for secondary flight feathers and inner tail feathers [3].

### 5.2. Mechanical properties

The mechanical properties of feather barbs depend on feather type, bird species and environmental conditions. Modulus and strength values from the literature are shown in table 2. Tensile stiffness values of the barb are slightly lower than the cortex values (modulus of approx. 2–4 GPa), but much higher than the medulla values (modulus of approx. 0.1−10 MPa). Differences in material properties between species and feather type are an indication of differences in structure, from the keratin conformational structure at the nanoscale to medulla/cortex structure at the microscale.

**Table 2.** Tensile tests till failure have been performed on the barbs of several species from different feathers and at varying environmental conditions. Ordering the test outcomes from least stiff to most stiff reveal three effects: (i) since factors affecting the stiffness of biological materials often have a concomitant effect on their strength, both of these properties show similar trends, (ii) tests performed at higher relative humidity decreased both stiffness and strength, indicating the importance of conducting mechanical characterization in environmentally relevant conditions, and (iii) barbs from downy feathers tend to have lower stiffness and strength, possibly as a result of a morphological difference, which may require a different testing paradigm to resolve fully. For material properties the standard error of the mean (s.e.m.) is reported across samples tested.

modulus (GPa)	strength (MPa)	species	feather type	RH (%)	temp. (°C)	strain rate (mm min^−1^)	reference
1.3 ± 0.2	79 ± 7	duck	downy	96	20	—	[98]
1.3+0.2	104 ± 15	goose	downy	73	23	—	[99]
1.7 ± 0.1	220 ± 11	penguin	downy	73	23	—	[100]
2.1 ± 0.2	166 ± 13	duck	downy	50	20	—	[98]
2.2 ± 0.2	148 ± 15	duck	downy	73	23	—	[99]
3.6+0.1	203 ± 9	chicken	pennaceous	50	20	1.27	[101]
4.6 ± 0.2	187 ± 8	chicken	pennaceous	65	21	18	[102]
—	281 ± 6	osprey	primary	—	—	8	[94]

Because so many structural parameters can be tuned to optimize mechanical properties, barb studies are complicated. For example, melanin rods in the barb were believed to increase resistance to abrasion and lower barb breakage; i.e. dark, melanized barbs are stronger than their light counterparts. However, even though darker barbs from an osprey (*Pandion haliaetus*) had a higher breaking force than lighter barbs, this difference could be explained by the position of the barb along the rachis [94]. Specifically, barbs closer to the bird’s body (which were primarily white) were less stiff than barbs farther away (which were primarily black). This coincides with keratin packing becoming more aligned and closer together in the proximo-distal direction along the rachis [94], showing how even the smallest hierarchical design scale drives feather properties.

Future research at the barb level should correlate barb morphology to function for a wide range of bird orders and connect this back to the micro- and nano-structure to map the phylogenetic relationship between structure and property. For example the barb of an emu contour feather is covered in small pits, a reticulate-foveate pattern, which serves to (i) improve the heat-insulating capacity of the feather by increasing the air layer in the plumage and scattering sunlight, and (ii) store moisture in the plumage to protect the body from excessive water loss [67]. Resolving how this barb structure relates to the variability in the amino acid sequence of the emu feather would connect structural observations over six orders of magnitude. Given this adaptation, how does ecotype affect barb shape, structure and properties? Do changes in barbules, or smaller structures, correlate with barb changes across species? What synergies are formed between the ramus (the launching point of barbules) and the rachis (the launching point of barbs)? Integrating all hierarchical design scales is an open challenge that needs to be resolved to advance our understanding of feathers and its role in the evolution of feathered dinosaurs and ultimately modern birds [103,104].

## 6. Rachis—centimetre scale

At first glance, the rachis only provides a platform that connects the aerodynamic vanes to a bird’s body. However, this viewpoint ignores the detailed structure that endows the rachis with its mechanical properties, and how minute changes in this architecture are reflective of a bird’s flying style and ecotype. This section focuses on the rachis, and how its structure–property relationship diversity enables a wide range of bird behaviours.

### 6.1. General structure

The rachis can be thought of as a fibre-reinforced, laminate composite tube encapsulating a foam-like interior with fibres systematically diverging into the rami from feather base to tip. This branching effectively tapers the rachis towards the tip [62]. Details of rachis structure, such as number of layers and fibre angle in each layer is thought to vary between species, depending on flying style, but universal features of the rachis architecture are synergistically built for optimal mechanical properties [105].

**Figure 8.**
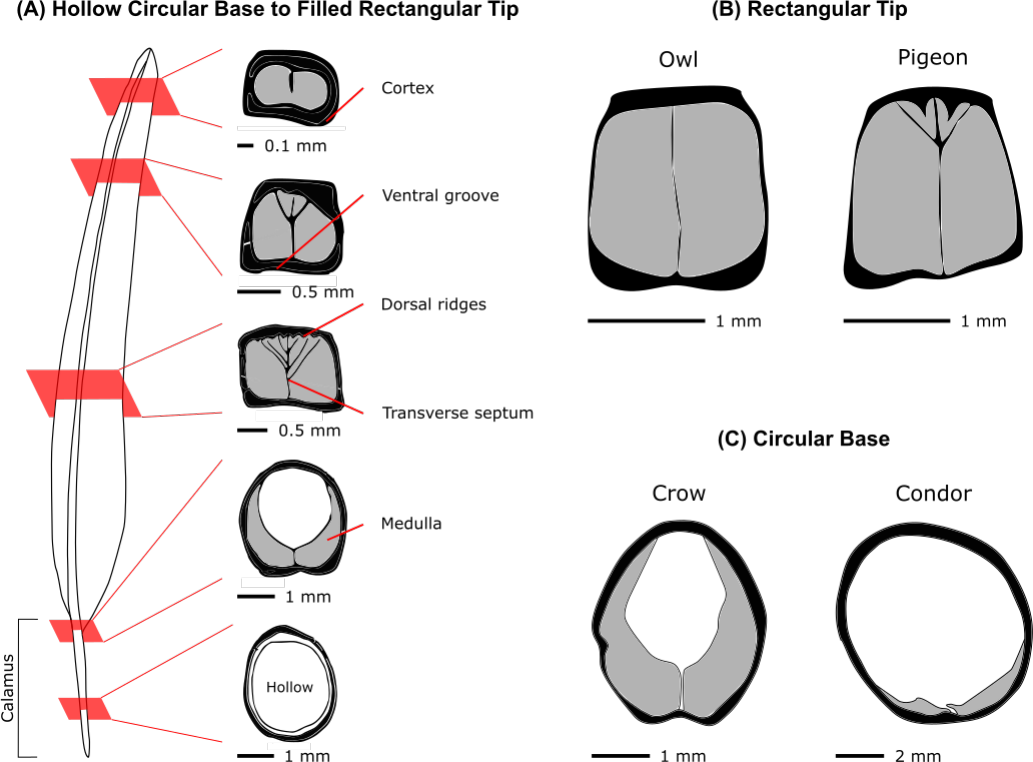
Rachis shape varies across feather length and between species to facilitate graded load bearing functionality. Species-specific variations have been noted, but not thoroughly studied. (A) The base of the rachis, which continues from the hollow calamus, is circular in shape while the tip is rectangular. The slow transition in shape is accompanied by the emergence of the medulla and the potential blossoming of the transverse septum, ventral groove and dorsal ridges. (B) Rectangular cross section near rachis midsection of owl and pigeon feathers showing the much more developed transverse septum and dorsal ridges of the latter. (C) Circular cross sections near the rachis base of crow and condor feathers illustrate marked interspecies differences. Adapted from [106–108].

The rachis cross-sectional shape is constructed to gradually change mechanical properties along the feather. The cross-section changes from circular to rectangular starting at about 20% shaft length (figure 8A). Since the rachis tapers off to reduce drag and save weight, this change in cross section helps tailor stiffness by counteracting the decrease in cross-sectional area, thereby maintaining the level of flexural rigidity required for flight in turbulent gusts throughout the entire feather [106]. Simultaneously, Young’s modulus of the cortex tends to increase along the rachis [109], from base to tip, as a result of keratin filaments becoming more tightly packed and aligning parallel to the rachis’s long axis, demonstrating how the feather’s hierarchical design spans all scales. Remarkably, flightless birds do not conform to this general trend, perhaps as a result of differences in the amino acid sequence of their feather β-keratin [110], showing how specific evolutionary pressures, such as those associated with the loss of flight, select different hierarchical feather design outcomes.

A cross section of the rachis reveals that the cortex of the dorsal and ventral surface tends to be thicker than the epicortex lateral walls so that dorso-ventral bending stiffness is greater than lateral bending stiffness [106], thereby helping resist the aerodynamic forces experienced in flight during downstroke. One exception is the outermost primary, which is fatter/wider than other feathers and therefore has greater flexural stiffness in the lateral rather than dorso-ventral direction. This may be to resist drag forces, the bulk of which are experienced by this leading edge feather [111].

The characteristics examined so far are observable across bird orders; however, other structural features identified in specific bird species remain understudied. For example, dorsal ridges occur when the dorsal cortex creates tiny bumps into the medulla. Another structure known as the transverse septum divides the medulla into two chambers and the ventral groove marks a concave region underneath the feather (figure 8A). These architectural details have the potential to fine-tune mechanical properties, but a systematic study across bird orders has yet to be conducted. Unlike the general rachis structure that appears to be similar in all birds, these features could vary depending on evolutionary constraints and play bigger driving roles in the feather’s hierarchical design and function than currently considered.

### 6.2. Species-specific structural variations

Rachis structure differences between species can occur for a variety of reasons such as flight style and migratory behaviour. For example, larger birds have relatively shorter and more flexible primary feathers than smaller birds to reduce stresses on the wing skeleton during take-off and landing [112,113]. These changes in structural and mechanical properties help accommodate large birds’ flying styles, which involve gliding or soaring since the power required for flapping flight increases more rapidly with mass than the available power provided by muscles (roughly 100 W kg^−1^) [114]. Compare these with the flying styles of smaller birds, which primarily use flapping or bounding, and therefore need feathers that will not deform extensively under high loads [112,113]. Furthermore, the elastic energy stored by bending in the rachis during downstroke may be converted into useful aerodynamic work at the end of the downstroke [115]. Due to the (putative) barbule and barb unidirectional valve flap function, this energy is probably not well stored during the upstroke. In fact, compressive strains on the dorsal surface of a feather during downstroke are more than double the tensile strain measured during upstroke [116]. Correspondingly, the aerodynamic force generated during downstroke is double that generated during upstroke [117].

The idea that flexural stiffness of the rachis is dictated more by geometry than by material properties has been proposed to explain the different flight styles between species [107]. For example, the values of keratin stiffness (Young’s modulus) do not vary significantly between barn owl and pigeons; however, the rachis of barn owls is far less structured, lacking dorsal ridges and a transverse septum through the medulla (figure 8B). Consequently, the barn owl’s rachis is less stiff in the dorso-ventral and rotary directions, which researchers hypothesize allows for the quick bending and twisting required during tight hunting manoeuvres in the air [107]. Compare this with the pronounced structure found in the rachis of pigeons, who beat their wings at high frequencies to become airborne and outmanoeuvre predators, thereby requiring stiff feathers to withstand large loads [107]. Marked structural differences can also be observed near the feather base of other species (figure 8C). The integration between the medullary pith and rachidial cortex is known to delay the onset of local buckling compared with hollow cylinders [55]; however, how different bird species modulate these features to control buckling behaviour—as seen in other biological structures (e.g. plant stems and animal quills) [76]—remains unknown. Even individuals of the same or similar species, but with differing migratory [118], sexual [119,120] or non-verbal communicative [121] behaviours demonstrate changes in rachis and feather shape that modulate stiffness. This points to the importance of more deeply integrating movement ecology [122] and sexual selection pressures in mechanistic analyses to understand feather function evolution.

Future work at the rachis level should focus on systematic investigation correlating structure with properties and ecotype. First, researchers should identify which birds have dorsal ridges, ventral grooves, traverse septa and any other morphological features that endow structural function. Second, studies should test how physical properties arise from these hierarchical structures to truly understand the mechanisms underpinning the observed differences. Because interspecies comparisons cannot be performed without considering morphological, behavioural and environmental influences on mechanical properties, these comprehensive studies will require knowledge on both phylogenetic trees and their associated statistics, selection pressures and the mechanics of composite materials. This understanding will prove useful not only when studying the level just above the completed feather, the wing (electronic supplementary material, §1), but also when seeking inspiration from the feather’s many functions (see electronic supplementary material, §§2 and 3 for detailed examples).

## 7. Bioinspiration and research outlook

**Figure 9.**
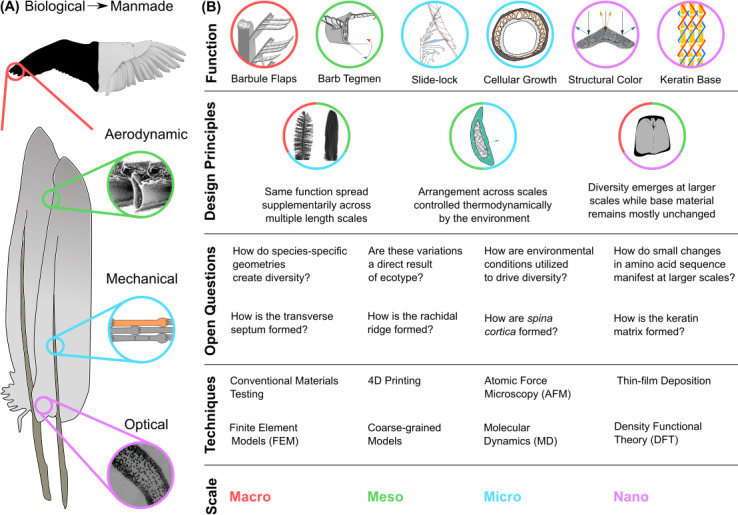
Bird feathers follow specific design principles spread across multiple length scales to fulfil a variety of functions. Man-made designs will benefit from fully understanding and translating these principles. (A) As the biomimetic leap is made from biological to man-made designs—whether this be for the aerodynamic surface continuity via locking between two feathers through lobate cilia at the mesoscale; the mechanical prevention of crack propagation between fibres at the microscale; or the optical structural colouring of melanin films at the nanoscale—several key principles need to be integrated. (B) Despite identifiable features being present at every length scale, most functions are spread across scales in an auxiliary manner. For example, wing planform stability during upstroke/downstroke is maintained through (putative) barbule flaps, barb tegmens, the slide-lock system between barbules as well as between overlapping flight feathers, among other mechanisms; in this way, the failure of one feature does not compromise the whole system. Furthermore, the distribution of functions across scales is usually controlled thermodynamically with the structure being largely dictated by selection pressures in the bird’s environment. This allows for immense diversity as structures change across scales, while the base material remains mostly unchanged. To fully understand the implications of these design principles in the bird feather, several open questions (both general and specific) should be addressed. We list a few of the examples outlined in the text. This will involve both experimental and computational techniques that can probe every order of magnitude, as well as theories that bridge the exceptionally wide range of scales. Adapted from [3,18,36,45,80,107]. Reprinted from [84,87,123–125].

This review shows there is no shortage of inspiration from feathers as potential designs can be enkindled at every length scale and from many different functions (e.g. locomotion, thermoregulation, waterproofing, coloration and sound generation are particularly enticing for bioinspired applications) (figure 9A). Researchers across disciplines have developed several approaches inspired by nature including: aerodynamic noise reduction inspired by owl wings [126]; aerial robotic design inspired by wing morphing [123,127]; insulation materials inspired by downy feathers [99]; composite and textile production inspired by rachis morphology [105,128]; and even optical devices inspired by self-assembled melanosomes [129]. Three principal approaches, each with distinct advantages and limitations, translate findings in natural materials to man-made designs: (i) biohybrid tactics where biological components are integrated into the design, e.g. incorporating real feathers in products ranging from pillows to aerial robots [123]; (ii) biomimetic methods where structures are replicated as closely as possible in the design, e.g. mimicking lobate cilia structures to make directional Velcro [87,88]; (iii) bioinspired approaches where the design principles distilled from the natural structure are harnessed for engineering novel structures, e.g. combining structural and pigmentary colour mechanisms to produce novel visuals [130,131]. The boundaries between these design approaches can be blurry, and the optimal approach depends on the desired application, current engineering design and manufacturing constraints. By examining how researchers have previously designed feather-inspired solutions, we identify key knowledge gaps to motivate the next wave of feather-inspired designs and research.

### 7.1. Current feather-inspired designs

At the smallest level of organization, researchers have used a biohybrid approach to harness β-keratin as a basis for creating natural, injectable or preformed hydrogels. Even after an intense process to isolate β-keratin from its natural tissue, the nanostructure remains an integral part of the hydrogel. These hydrogels are posited for use in cell delivery in tissue engineering and regenerative medicine [132]. Similar biohybrid approaches, where feathers are used as the fundamental building material, produce biocomposites where a polylactic acid (PLA) matrix enshrouds feather barbs that act like fibres [102,133]. Due to the abundance of feathers from poultry waste, biohybrid approaches offer the advantage of assimilating the feather as a material for construction in a circular economy [134].

Biomimetic approaches have been used to make lighter-weight, stronger carbon fibre-reinforced polymer (CFRP) composites mimicking a feather’s fractal architecture from the rachis to the barbs, the barbules and the barbule outgrowths [128]. Aerial robots such as Roboswift [135] and LisHawk [136], which can skilfully morph their wing shapes midflight like birds, are other examples of successful biomimetic implementation. Biomimetic approaches are not affected by shortages caused by limited supplies of biological materials, and can use existing processing techniques to achieve scalability and reliability.

Bioinspired design is perhaps the most assiduous approach because it requires deep knowledge of the design principles of nature before starting to prototype. However, it is also the most holistic method, because the design principles can be abstracted and transmuted to adapt to a given problem’s current engineering constraints [6,137]. In this way, strong human-made materials can be thoughtfully incorporated into nature’s hierarchical structures; for example, in the production of a 3D printed structure made from acrylonitrile butadiene styrene (ABS) plastic that mimics the unidirectional permeability and repairability of barbules, but not their shape [84]. These deployable structures have potential for use in next-generation chain mail armours and foams.

Significant progress is still needed for harnessing the potential of bioinspired design. For centuries, humans have used downy feathers for thermal insulation because of down’s unsurpassed warmth-to-weight ratio, compressibility and compression recovery [138,139]. While alternative synthetic insulation materials are cheaper, retain their insulation and dry quicker, none can match the warmth-to-weight ratio and lifespan of downy feathers [140]. Mechanical, thermal and structural measurements of down are regularly published with the expectation that biomimetic insulation materials should possess similar properties [99], but it may be that the most practical insulating material does not have the same properties as down. Rather, designs should aim to replicate underlying principles of nature, even if composed of entirely different materials.

### 7.2. Design principles and knowledge gaps for future feather-inspired research

The recent discovery of adding a polymer matrix between carbon nanotubule fibres to create a tough material [55] has been around for approximately 150 million years as a feather design principle. Indeed, the cortex of the rachis consists of bundles of 3 nm diameter β-keratin filaments embedded in an amorphous keratin matrix (figure 6B) that produce the exceptional feather strength-to-weight ratio [108,141]. What other hierarchical and multi-functional design principles have we found after systematically surveying the hierarchical architecture of feathers (figure 9B)?

At the smallest length scale, β-keratins form filaments and matrix from the *same* amino acid chain (figure 3) [17]. This design principle constitutes a paradigm shift for materials science engineering that defines a composite material as a mixture of two or more components [142]. Issues currently plaguing engineered composites include fibre pull-out, fibre matrix delamination and long-term fatigue [143,144]. Building composites that are strongly connected from the beginning of the manufacturing process could help avoid such failures by harnessing compatibility at the molecular level (covalent versus hydrogen bonding). To achieve this goal, we first must address the knowledge gap of how feather keratin’s two terminal ends fold.

At the cellular level, inspiration can be drawn from the process of cell differentiation that creates a multi-functional feather structure. The self-assembly steps in this formation process are thermodynamically favourable, and therefore do not require explicit cellular control or energy expenditure. This design principle is evident in the phase separation of the medulla or melanin deposition in the barbules, processes that have sub-micrometre to nanometre levels of precision and create coloration in bird feathers, indicating that slight alterations in genetic programming, initial and boundary conditions, and/or development environment (temperature, pH, diet, etc.) can entropically drive very different results.

Furthermore, cells act as an ‘active pre-programmed’ material that differentiates into various cell types with different, but thermodynamically favoured, multi-functional properties [145]. For example, supportive cells play a pivotal role in carving out the barbule structure of feathers, but are removed once genesis is complete. The cell differentiation design principle is poised to inspire new four-dimensional (4D) hierarchical printing technology where programmable materials can precisely build up large hierarchical structures by reacting to environmental factors such as temperature and humidity [146].

Filling in the knowledge gaps about cellular development, could potentially launch a revolution in multi-functional additive manufacturing. For example, cells differentiate along a helical path to form pennaceous feathers, but how do they merge at the rachidal ridge? How do cells on the dorsal and ventral sides remain as fibres, while cells near the epicortex decompose until only macrofibrils remain? How do cells control their surface roughness during differentiation, and why does this vary between bird orders? What is the medulla made of and how does it form? Answering these questions can help us incorporate structural principles such as cortex toughening (brick-bridge-mortar), medulla structural colour, hydration-induced repair and barbule fastening mechanisms into human-made designs.

Investigations at the larger scale of the barb and rachis are at a point where basic structural features are well known. Therefore, progress in these areas could focus on the hierarchical patterning such as the reticulate-foveate pattern in emu barbs or the transverse septum of pigeon rachis, and how these vary across bird orders to furnish different functions such as spontaneous de-wetting of aquatic birds. Relating these unique features to specific functionalities imposed by evolutionary and environmental constraints is crucial to identify and link design concepts. This requires a deep understanding of both the biology behind phylogenetic relations and evolutionary pathways, and the biophysics and biomechanics underpinning emergent material properties. In other words, it is important to know the definition of plasticity in both the phenotypic and mechanistic sense. Specific structural features that should be investigated at these levels include: (i) the branching of the barb from the rachis, as this connects barbule morphology at the micrometre level to feather aerodynamics at the sub-millimetre to metre level, and (ii) the effect that barb and rachis shape, as opposed to keratin material properties, have on controlling feather mechanical properties.

There is a clear effort by nature to spread the feather’s multiple functions supplementarily across scales. For example, considering both barbule unidirectional valves (µm) and barb branching angle (mm) in one study can resolve the disparity in force generated during upstroke versus downstroke (or rebuke the valve hypothesis). The new insights can advance the design of flapping winged robots. We have also observed that modulation of keratin orientation (nm) can make the rachis (cm) stiffer from base to tip. Fibre orientation is already used in various human-made composites, but could be further utilized to create graded changes in mechanical properties tailored to meet local constraints. The combination of medullary foam (µm) and a transverse septum (cm) to provide buckling resistance while contributing little to feather weight is another exciting design feature. Connecting just two spatial scales in feather structure as a first step, rather than integrating the whole hierarchy, can accelerate structural design innovation.

The final avenue for bioinspiration comes from the integration of all length scales into one complete, holistic design. This is no small task as the tools and equipment used to investigate proteins at the nanometre scale differ from those used to study wing planforms at the millimetre to metre scale. Different methods offer distinct advantages and suffer from unique weaknesses, thereby a more thorough investigation is only possible by combining approaches. Additive manufacturing techniques, such as 4D printing and thin-film deposition, offer a bottom-up approach reminiscent of biological growth, but current methods do not offer nanometric control while constructing large, usable structures (cm–m scale). In comparison with feathers with their 10^9^ orders of magnitude of integration, conventional 3D printing technology can tie together 10^5^ orders of magnitude for objects on the scale of a metre with approximately 10–250 µm resolution steps [147,148] and inches towards 10^6^ for objects on the scale of tens of mm size with approximately 100 nm feature size [149]. The superiority of graded biological structures [150] to additive manufacturing, with their finer resolution and increased extension to thousand times larger scales is heightened by the composite nature of feathers, requiring simultaneous deposition of several materials with various mechanical properties; something man-made techniques still struggle with [6,137].

**Figure 10.**
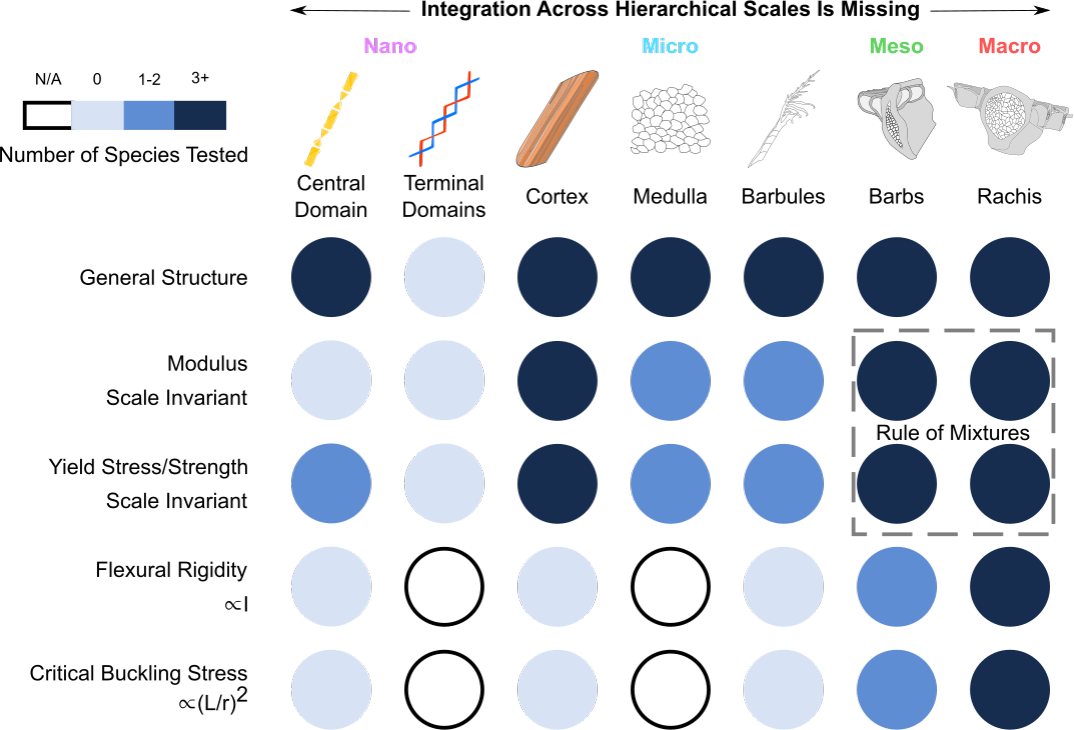
Characterization of feather material and structural properties has focused on large length scales and few species. The general hierarchical structure of feathers has been coarsely established across all length scales (the terminal domains of feather keratin being a notable exception), but limited information exists on key physical properties at each scale and how they cumulatively build up function across scales. For example, we found no studies (experimental nor computational) quantifying the molecular rigidity of the feather keratin filament formed from the central domain nor the matrix formed from the terminal domains. These values, and the orientation of these molecules, affects the elastic modulus, strength and anisotropy of the cortex [70,72–74,155,156], medulla [76,78] and barbules [157]. Although modulus does not depend on sample size, medullary foam modulus does depend on packing density $\rho_{medulla}$ as $E_{medulla}\propto E_{keratin}{\left(\rho_{medulla}/\rho_{keratin}\right)}^{n}$, where *n* is between 2 and 3. For the barb [102,158] and rachis [107,111,159,160], we expect the modulus and strength of these structures to be somewhere between their cortical and medullary components as dictated by the rule of mixtures. Specifically, when loaded under tension parallel to fibre orientation, $E_{barb}=V_{cortex}E_{cortex}+V_{medulla}E_{medulla}$, where $V_{cortex}$ and $V_{medulla}$ are the volume fractions of the cortex and medulla in the barb. A similar expression exists for the rachis. Since many of the structures found in the feather’s design are elongated beams, we also summarize measurements done on the flexural rigidity [78,107,112,161] (scales linearly with second moment of area I) and critical buckling stress [116,160,162] (scales quadratically with slenderness ratio L/r). These properties are not applicable (N/A) to non-beamlike structures such as the matrix terminal domains and the medulla. How all these substructures are modulated and assembled into a feather’s hierarchical architecture to fit individual functional needs of a species remains a critical question for future work.

Recently, a new era of computational materials science has begun, driven by interest in efficiently predicting the properties of new materials instead of explaining the properties of known materials. This paradigm shift has been accompanied with a growing interest in modelling materials using multi-scale modelling based on ground-up (*ab initio*) calculations and data-driven approaches such as machine learning, with little to no empirical knowledge, to describe larger and more complicated systems [151,152]. Feathers are an ideal system to study for this purpose since they exhibit a remarkable range of properties (refined over hundreds of millions of years via evolution) that would be highly desirable in various human-made applications. Density functional theory calculations, combined with molecular dynamics and finite elements, can be used to rapidly alter the digital twin of a feather’s hierarchical design and predict resulting properties. In the process this also has the potential to elucidate evolutionary pathways that have been previously favoured by natural selection. Furthermore, integrating these computational techniques with various imaging and experimental characterization tools, e.g. diffraction, spectroscopy, electron/atomic force microscopy and microelectromechanical testing, enables integral exploration of hierarchical materials across all relevant time and length scales. This serves as the basis for the emerging field of materiomics [153,154]. However, theories connecting scales remain lacking. This is in part because the design space for these hierarchical materials is so vast—several orders of magnitude larger than the design space of conventional, engineered materials. One way to more effectively begin exploring this space is by comparing the feather’s hierarchical structure across the avian phylogenetic tree. This remains an open challenge (figure 10). However, by combining the experimental techniques of materials science with phylogenetic tree analysis, it becomes possible to identify immutable design principles found in all feathers. It will also highlight adaptation specific structural features responsible for the remarkable diversity between feathers. This makes hierarchical materials, including feather-inspired designs, the nexus of materials science and biology as both fields will play key roles in resolving the structure–property–function relationships of these materials.

### 7.3. Concluding synthesis

The feather is a complex, biological structure that endows birds with a myriad of properties. This grand complexity is hierarchically constructed from a simple building block present in all feathers of all birds: feather keratin. Therefore, the design space of bird feathers is expansive not because of variations in chemical composition, but due to the multiple scales at which ecological and physical pressures have imposed their structural alterations during the process of evolution. These architectural changes occur naturally during development; i.e. the creation of these structures is thermodynamically favourable and requires no explicit cellular control. In this way, birds not only achieve their vast diversity of feather types, but also stupendous multifunctionality. Our review shows investigating these structure–property–function relationships will require a multi-disciplinary thrust to develop numerical models, characterization techniques and manufacturing methods capable of capturing and replicating the hierarchical multiphysics of the bird feather.

## Data Availability

Supplementary material is available online at [163].
